# Correction: Molecular and materials design for efficient solar energy conversion: a review of photochemical technologies

**DOI:** 10.1039/d6ra90047j

**Published:** 2026-05-21

**Authors:** Abdulrahman A. Alsimaree, Saeed S. Samman, Abdulaziz M. Almohyawi, Hatem M. Altass, Jan Mohammad Mir, Saleh A. Ahmed

**Affiliations:** a Department of Chemistry, College of Science and Humanities, Shaqra University Shaqra Saudi Arabia; b Department of Basic Science and Technologies, Applied College, Taibah University 42353 Madina Saudi Arabia; c Department of Chemistry, Faculty of Science, Umm Al-Qura University 21955 Makkah Saudi Arabia saahmed@uqu.edu.sa saleh_63@hotmail.com; d Multidisciplinary Chemistry Laboratory, Department of Chemistry, Islamic University of Science and Technology Kashmir 192122 India mirjanmohammad@gmail.com

## Abstract

Correction for ‘Molecular and materials design for efficient solar energy conversion: a review of photochemical technologies’ by Abdulrahman A. Alsimaree *et al., RSC Adv.*, 2026, **16**, 5864–5876, https://doi.org/10.1039/D5RA09833E.

The authors regret the omission of the funding statement from the above-named manuscript. The statement is as follows:

Funding statement: This research work was funded by Umm Al-Qura University, Saudi Arabia under grant number: 26UQU4320545GSSR03.

The Royal Society of Chemistry apologises for these errors and any consequent inconvenience to authors and readers.

